# The Thermodynamics of the Van Der Waals Black Hole Within Kaniadakis Entropy

**DOI:** 10.3390/e26121027

**Published:** 2024-11-27

**Authors:** Adam Z. Kaczmarek, Yassine Sekhmani, Dominik Szczȩśniak, Javlon Rayimbaev

**Affiliations:** 1 Institute of Physics, Faculty of Science and Technology, Jan Długosz University in Czestochowa, 13/15 Armii Krajowej Ave., 42200 Czestochowa, Poland; adamzenonkaczmarek@gmail.com; 2 Center for Theoretical Physics, Khazar University, 41 Mehseti Street, Baku AZ1096, Azerbaijan; sekhmaniyassine@gmail.com; 3 Département de Physique, Equipe des Sciences de la Matière et du Rayonnement, ESMaR, Faculté des Sciences, Université Mohammed V de Rabat, Rabat 10000, Morocco; 4 Institute of Fundamental and Applied Research, National Research University TIIAME, Kori Niyoziy 39, Tashkent 100000, Uzbekistan; javlon@astrin.uz; 5 Faculty of Computer Engineering, University of Tashkent for Applied Sciences, Str. Gavhar 1, Tashkent 100149, Uzbekistan; 6 Faculty of Technics, Urgench State University, Kh. Alimjan Str. 14, Urgench 221100, Uzbekistan; 7 Faculty of Pedagogy, Shahrisabz State Pedagogical Institute, Shahrisabz Str. 10, Shahrisabz 181301, Uzbekistan

**Keywords:** entropy, Kaniadakis entropy, non extensive entropy, black holes, thermodynamics, information paradox

## Abstract

In this work, we have studied the thermodynamic properties of the Van der Waals black hole in the framework of the relativistic Kaniadakis entropy. We have shown that the black hole properties, such as the mass and temperature, differ from those obtained by using the the Boltzmann–Gibbs approach. Moreover, the deformation κ-parameter changes the behavior of the Gibbs free energy via introduced thermodynamic instabilities, whereas the emission rate is influenced by κ only at low frequencies. Nonetheless, the pressure–volume (P(V)) characteristics are found independent of κ and the entropy form, unlike in other anti-de Sitter (AdS) black hole models. In summary, the presented findings partially support the previous arguments of Gohar and Salzano that, under certain circumstances, all entropic models are equivalent and indistinguishable.

## 1. Introduction

Black holes (BHs) are one of the most intriguing entities in modern physics, as they are crucial for understanding the fundamental relationship between gravity and the quantum world, including the unification of gravity, quanta, and statistical physics [1,2]. From this perspective, the fact that BH emits (Hawking) radiation due to the evaporation process provides the pivotal concepts of Hawking temperature and Bekenstein entropy associated with the BH horizon [3,4]. In fact, the process of evaporation appears to be non-unitary, leading to the famous black hole (or information loss) paradox [2,5]. In this manner, the thermodynamic properties of BH pose serious challenges, even without explicitly invoking quantum field theory. In particularly, the maximal information falling into a BH can be expressed as entropy [1,6,7,8,9]. Moreover, there are some intriguing aspects regarding the entropy of a BH, when one considers the holographic principle as a guiding physical postulate [10,11]. According to this principle, the black hole horizon can store information in the form of a hologram. In this context, anti-de Sitter (AdS) black holes have been the subjects of important studies in recent decades, mainly due to the so-called AdS/CFT correspondence [12]. The AdS/CFT conjecture states that the gravity sector involving the non-compact AdS space can be interpreted as a thermal field theory from an asymptotic viewpoint. Another interesting finding associated with the AdS/CFT is the exploration of first-order phase transitions in black holes [13,14,15]. These transitions have been found to be analogous to those observed for liquid–gas transitions from thermodynamics and chemistry [16,17,18]. In this framework, the negative cosmological constant is interpreted as pressure [19,20], leading to an enhanced phase space where the equation of state P=P(V,T) allows for the study of the critical behavior of AdS black holes [20,21]. The analogy between black holes and real gases is further extended by the Van der Waals (VdW) black hole model, which incorporates critical behavior and phase transitions similar to those observed in VdW fluids [22]. This model provides a richer thermodynamic description, where the pressure–volume–temperature relationship mimics the VdW equation for real fluids [22].

However, the standard description of BH entropy linked with the Boltzmann–Gibbs (BG) viewpoint gives rise to rather unconventional scaling. The holographic principle indicates that the BG entropy $S_{BG}$ of a (3+1)-dimensional black hole is proportional to its area $L^{2}$, rather than its volume $L^{3}$. Similarly, for strongly quantum-entangled *d*-dimensional systems, the area law states that $S_{BG}$ is proportional to lnL if d=1, and to $L^{d-1}$ if d>1, rather than $L^{d}$ (d≥1). Consequently, the additiveness of the thermodynamic entropy of a *d*-dimensional system no longer holds true [23]. This issue led to the search for generalizations and nonadditive approaches to the statistical description of thermodynamics, introducing concepts such as Barrow or Tsallis statistics within the BH thermodynamics [24,25,26,27]. In this context, Renyi and other entropies have been studied recently as well [28,29,30,31,32,33]. For a review of the different entropies, we refer the reader to [34].These possibilities of describing statistics in relativity are connected to thermodynamic conjecture, which treats spacetime as an ordinary thermal system, with Einstein equations viewed as a equivalent to the laws of thermodynamics [35,36,37]. In this context, Kaniadakis statistics (known as *K*-statistics) are of particular interest, due to them being a coherent relativistic statistical theory, inspired by the symmetry of the Lorentz group [38,39,40,41]. The Kaniadakis approach has gained significant attention, especially in cosmology, for its applicability to the slow-roll inflation mechanism [42] and the PeV neutrino tension [43]. Interestingly, one of the most significant achievements in favor of using Kaniadakis statistics is its successful explanation of the non-Boltzmannian spectrum of cosmic rays. Specifically, the observed power-law tails in the cosmic ray spectrum are a direct consequence of relativistic effects, which are correctly predicted by the κ-statistics framework [44]. As for the BH physics, Kaniadakis statistics pose the ability to accurately describe the Bekenstein–Hawking area law [45]. Therefore, there are compelling reasons to extend BG statistics for black hole physics, with the Kaniadakis approach being particularly favored for its inherent relativistic essence [38,39,40,41]. This is another advantage of κ-entropy as, contrary to other entropic models, such as Tsallis entropy or Renyi entropy, it is actually derived from a relativistic generalization of the Boltzmann–Gibbs framework [44]. It retains important properties of classical thermodynamics, while allowing for nonadditive corrections. This makes it particularly suitable for black hole thermodynamics, where quantum gravitational effects can lead to non-standard statistical distributions [23].

Motivated by the interesting interplay between BH chemistry concept and nonadditive statistics in BH physics, the following work aims to study main thermodynamic aspects of VdW BH. Our primary focus is on the relevance of Kaniadakis statistics to Van der Waals BH physics, where they offer a novel perspective on entropy and thermodynamic stability. However, we recognize that this alternative statistical model could also be of interest within the gauge–gravity correspondence community. The AdS/CFT duality, which connects black holes in anti-de Sitter (AdS) space with thermal states in conformal field theory, provides a natural setting for examining the effects of modified entropy models on both sides of the duality [12,46]. Thus, while our findings are related to black hole thermodynamics, they may also provide useful insights for researchers studying AdS/CFT. The outline of the present work is as follows: in Section 2, we present the description of VdW BH and basic thermodynamic quantities in the κ-statistics regime. In Section 3, we discuss the thermodynamic stability, revealing the local stability and the Gibbs free energy. After that, an examination of the energy emission rate is carried out in Section 4. Finally, Section 5 summarizes and draws conclusions of the work presented herein.

## 2. Kaniadakis Statistics and Van Der Waals Black Hole Geometry

The starting point in the description of Van der Waals BH is the modified line element of an AdS black hole in the following form [22,47]:

(1) $$\begin{array}{c} ds^{2}=-f\left(r\right)dt^{2}+f{\left(r\right)}^{-1}dr^{2}+r^{2}d\theta^{2}+r^{2}\sin^{2}\left(\theta \right)d\phi^{2}, \end{array}$$

where the radial function takes form:

(2) $$\begin{array}{c} f\left(r\right)=2\pi a-\frac{2M}{r}+\frac{r^{2}}{l^{2}}\left(1+\frac{3b}{2r}\right)-\frac{3\pi ab^{2}}{r(2r+3b)}-4\frac{\pi ab}{r}\log\left(\frac{r}{b}+\frac{3}{2}\right), \end{array}$$

where the magnitude of the intramolecular forces and molecular pressure are a>0 and b>0, respectively [22,48]. It is essential to add that a VdW black hole, as a solution of the Einstein equations, satisfies the energy conditions for smaller pressures *P* while being violated as *P* increases [22]. For an analysis of a VdW BH in higher dimensions, we refer to [49]. Notably, for $a=\frac{1}{2\pi }$ and b=0, the metric reduces to the usual Schwarzschild AdS line element. One can view the VdW BH as a thermodynamic generalization of the Schwarzschild black hole. Moreover, in that scenario, pressure can be associated with the cosmological constant Λ via [6,22]:

(3) $$\begin{array}{c} P=\frac{3}{8\pi l^{2}}=-\frac{\Lambda }{8\pi }. \end{array}$$

We note that pressure *P* can be understood as a energy–momentum tensor associated with the studied spacetime [8]. From this, it is possible to derive an equation of state of the form P=P(V,T) for a given black hole. By drawing analogies between the BH temperature, volume, and pressure with those of a fluid, a direct mapping to the corresponding fluid equation of state can be established [8,49].

Thus, the metric (1) is the extended version of the AdS BH spacetime. In order to carry out an investigation in the extended phase space, we also introduce thermodynamic volume in the following manner:

(4) $$\begin{array}{c} V={\left(\frac{\partial M}{\partial P}\right)}_{S,Q}=\frac{2}{3}r_{h}^{2}\left(3b+2r_{h}\right), \end{array}$$

where $r_{h}$ is a horizon radius. Then, mass can be obtained from f(r)=0:

(5) $$\begin{array}{c} M=\frac{1}{6}\pi \left[\frac{a(-9b^{2}+18br_{h}+12r_{h}^{2})}{3b+2r_{h}}-12ab\log\left(\frac{r_{h}}{b}+\frac{3}{2}\right)+4Pr_{h}^{2}\left(3b+2r_{h}\right)\right]. \end{array}$$

It is worth remarking that, for pressure expressed as a cosmological constant, Λ mass (5) possesses a new interpretation. Specifically, one can treat the Schwarzshild mass of a black hole as a enthalpy, which leads the internal energy to be lowered relative to the mass by a positive *P* [19,21]. Additionally, it is known that Van der Waals black holes satisfy the first law of the BH thermodynamics [22,50]:

(6) $$\begin{array}{c} dM=TdS+\Phi dQ+VdP, \end{array}$$

as well as the Smarr relation:

(7) $$\begin{array}{c} M=2(TS-VP)+\Phi Q, \end{array}$$

where

(8) $$\begin{array}{c} T={\left(\frac{\partial M}{\partial S}\right)}_{P,Q},\;\;\;\Phi ={\left(\frac{\partial Q}{\partial S}\right)}_{S,P}. \end{array}$$

Specifically, explicit calculations of the Smarr relation for the Van der Waals black hole have been carried out in [51]. The Bekenstein–Hawking entropy of the VdW BH according to the standard Boltzmann–Gibbs statistics is:

(9) $$\begin{array}{c} S_{BH}=\frac{A_{BH}}{4}=\pi r_{h}^{2}. \end{array}$$

However, as already mentioned in the introduction, BG statistics may not be an appropriate way to describe the thermodynamic properties of a black hole, proving the need for viable forms of entropy. In what follows, Kaniadakis statistics leads to the relativistic notion of entropy. Please note that the relativistic nature of Kaniadakis statistics is associated with the inclusion of the relativistic relation between energy and momentum, as well as possessing the necessary relativistic invariance [38,39,40,41].

In detail, according to the Kaniadakis statistics, the entropy Formula (9) is modified and takes the following form [40,52]:

(10) $$\begin{array}{c} S=\frac{1}{\kappa }\sinh\left(\kappa S_{BH}\right), \end{array}$$

being nonadditive in its nature. The κ parameter is a deformation parameter that quantifies deviations from standard Boltzmann–Gibbs statistics, allowing the entropy to model systems with relativistic or nonadditive behaviors. When κ=0, Kaniadakis entropy reduces to the traditional Boltzmann–Gibbs form, indicating standard behaviour. As κ deviates from zero, the entropy incorporates power-law tails [41,44]. Note that modified entropy is a monotonically increasing function of $S_{BH}$ and, as a consequence, also a function of horizon radii $r_{h}$. Moreover, for the κ→0, standard Bekenstein–Hawking entropy is recovered. It is worth adding that since the function is even, we can focus on the κ>0 without losing generality [37]. From area law and expression for κ entropy, we obtain an inverse:

(11) $$\begin{array}{c} r_{h}=\frac{\sqrt{\frac{\sinh^{-1}\left(\kappa S\right)}{\kappa }}}{\sqrt{\pi }}, \end{array}$$

which will be used to study how Kaniadakis entropic description influences the properties of the Van der Waals black hole solution. Now, the mass of a black hole (5) can be expressed as a function of entropy (*S*):

(12) $$\begin{array}{cc} M(S) & =\frac{\sqrt{\pi }a\left(-3\pi b^{2}\kappa +6\sqrt{\pi }b\kappa \sqrt{\frac{\sinh^{-1}\left(\kappa S\right)}{\kappa }}+4\sinh^{-1}\left(\kappa S\right)\right)}{6\sqrt{\pi }b\kappa +4\kappa \sqrt{\frac{\sinh^{-1}\left(\kappa S\right)}{\kappa }}}-2\pi ab\log\left(\frac{\sqrt{\frac{\sinh^{-1}\left(\kappa S\right)}{\kappa }}}{\sqrt{\pi }b}+\frac{3}{2}\right)\\ & +\frac{2P\sinh^{-1}\left(\kappa S\right)\left(3b+\frac{2\sqrt{\frac{\sinh^{-1}\left(\kappa S\right)}{\kappa }}}{\sqrt{\pi }}\right)}{3\kappa }. \end{array}$$

This mass function actually contains different limits, leading to the different BH mass relations. For example, in the BG limit of Kaniadakis statistics (κ→0), the usual mass–entropy relationship of VdW BH is recovered:

(13) $$\begin{array}{c} M\left(S\right)=\frac{3\pi a\left(-3\pi b^{2}+6\sqrt{\pi }b\sqrt{S}+4s\right)-12\pi^{3/2}ab\left(3\sqrt{\pi }b+2\sqrt{S}\right)\log\left(\frac{\sqrt{S}}{\sqrt{\pi }b}+\frac{3}{2}\right)+4Ps{\left(3\sqrt{\pi }b+2\sqrt{S}\right)}^{2}}{6\left(3\pi b+2\sqrt{\pi }\sqrt{S}\right)}. \end{array}$$

**Figure 1:**
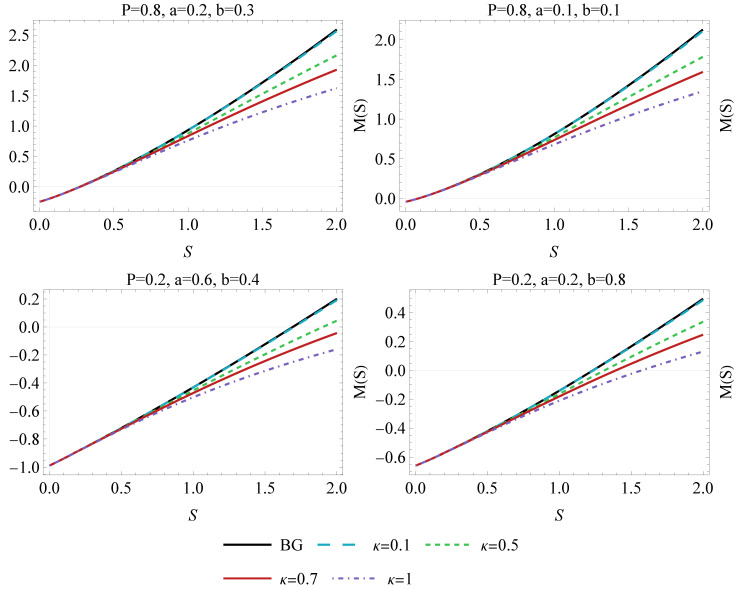
The mass parameter *M* as a function of entropy *S* for a different values of the κ parameter. The Boltzmann–Gibbs (BG) characteristics are marked by a black line (κ=0). The diagrams have been drawn for different pressure values, and VdW constants *a* and *b*.

The behaviour of the mass as a function of entropy has been plotted in Figure 1. Initially, the mass of a BH may be negative and thus unstable. However, once the entropy is increased, it will eventually reach positive values, with the rate of increase being affected by the values of κ, i.e., the lower the κ gets, the more rapid the increase. While the Kaniadakis entropy has no impact on the early stages, it significantly affects the final growth rate of a mass function. Note that similar behavior has been observed for a charged AdS black holes within the κ-statistics approach [52].

On the other hand, from the first law of BH thermodynamics (6) and Equation (12), the thermal radiation of a VdW BH reads:

(14) $$\begin{array}{cc} \; & T(S)=\\ \; & \frac{2\left(\pi^{3/2}b\kappa^{2}\left(a+9b^{2}P\right)\sqrt{\frac{\sinh^{-1}\left(\kappa S\right)}{\kappa }}+\sinh^{-1}\left(\kappa S\right)\left(\pi a\kappa +21\pi b^{2}\kappa P+16\sqrt{\pi }b\kappa P\sqrt{\frac{\sinh^{-1}\left(\kappa S\right)}{\kappa }}\right)+4P\sinh^{-1}{\left(\kappa S\right)}^{2}\right)}{\kappa^{2}\sqrt{\pi \kappa^{2}S^{2}+\pi }\sqrt{\frac{\sinh^{-1}\left(\kappa S\right)}{\kappa }}{\left(3\sqrt{\pi }b+2\sqrt{\frac{\sinh^{-1}\left(\kappa S\right)}{\kappa }}\right)}^{2}}. \end{array}$$

**Figure 2:**
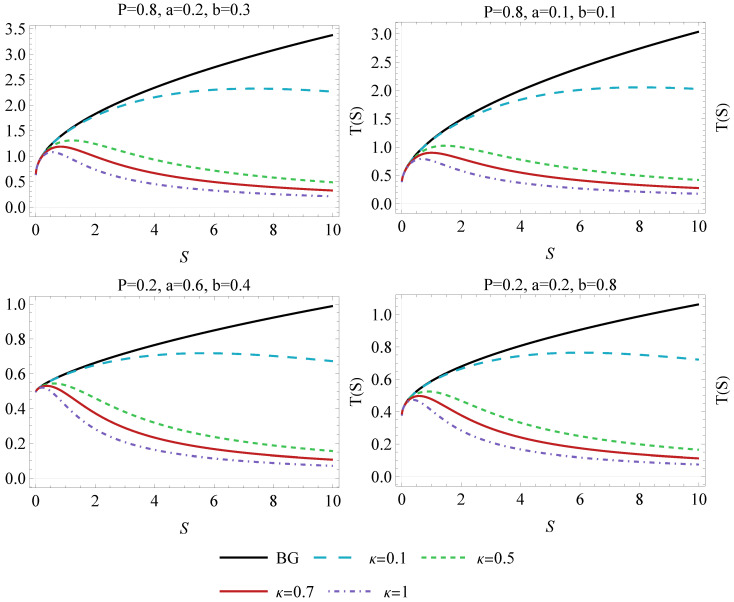
Thermodynamic temperature *T* as a function of entropy *S* for different values of the κ parameter. The diagrams have been plotted for different values of *P*, *a* and *b*. The behavior is compared to the BG entropy (κ=0).

The T(S) relation is presented in the Figure 2, where different choices of the BH parameters (a,b,P) are taken into account. This graphical analysis reveals that only κ→1 changes the behavior in a notable way. Interestingly, for some values of *S*, the radiation temperature reaches its peak $T_{crit}$. The peak shifts towards higher entropies as the Kaniadakis statistics become more relevant (i.e., κ increases). It is worth remarking that quantities modified by the Kaniadakis notion of entropy still satisfy the first law (6) and Smarr (7) relations, as was already pointed out by Luciano and colleagues [52].

## 3. Thermal Stability

### 3.1. Heat Capacity

In order to investigate the stability of the Van der Waals BH system, and see how Kaniadakis entropy affects it, one needs to analyze its heat capacity *C*. Positive values of *C* are associated with the stable region, while negative ones can suggest the unstable region. Additionally, potential divergences in the heat capacity may shed light on phase transitions and their interpretation [6,8,53,54]. In what follows, the heat capacity of a black hole can be defined as [53,55]:

(15) $$\begin{array}{c} C=\frac{\frac{\partial M}{\partial S}}{\frac{\partial^{2}M}{\partial S^{2}}}=\frac{T}{\frac{\partial^{2}M}{\partial S^{2}}}, \end{array}$$

and the Kaniadakis statistics of the VdW black hole read ($C_{0}=T$):

(16) $$\begin{array}{c} C=\frac{C_{0}}{C_{1}+C_{2}+C_{3}}, \end{array}$$

where

(17) $$\begin{array}{cc} C_{1} & =8P\kappa^{2}S\sinh^{-1}{\left(\kappa S\right)}^{2}\left(11\sqrt{\pi }b+2\sqrt{\frac{\sinh^{-1}\left(\kappa S\right)}{\kappa }}\right),\\ C_{2} & =\kappa \sinh^{-1}\left(\kappa S\right)(2\pi a\kappa^{2}S\left(5\sqrt{\pi }b+2\sqrt{\frac{\sinh^{-1}\left(\kappa S\right)}{\kappa }}\right)+162\pi^{3/2}b^{3}\kappa^{2}PS+180\pi b^{2}\kappa^{2}PS\sqrt{\frac{\sinh^{-1}\left(\kappa S\right)}{\kappa }} \end{array}$$

(18) $$\begin{array}{cc} & -36bP\sqrt{\pi \kappa^{2}S^{2}+\pi }-8P\sqrt{\kappa^{2}S^{2}+1}\sqrt{\frac{\sinh^{-1}\left(\kappa S\right)}{\kappa }}),\\ C_{3} & =\pi \kappa^{2}(a\left(6\pi b^{2}\kappa^{2}s\sqrt{\frac{\sinh^{-1}\left(\kappa s\right)}{\kappa }}+b\sqrt{\pi \kappa^{2}s^{2}+\pi }+2\sqrt{\kappa^{2}s^{2}+1}\sqrt{\frac{\sinh^{-1}\left(\kappa s\right)}{\kappa }}\right) \end{array}$$

(19) $$\begin{array}{cc} & +27b^{2}P\left(2\pi b^{2}\kappa^{2}s\sqrt{\frac{\sinh^{-1}\left(\kappa s\right)}{\kappa }}-b\sqrt{\pi \kappa^{2}s^{2}+\pi }-2\sqrt{\kappa^{2}s^{2}+1}\sqrt{\frac{\sinh^{-1}\left(\kappa s\right)}{\kappa }}\right)). \end{array}$$

**Figure 3:**
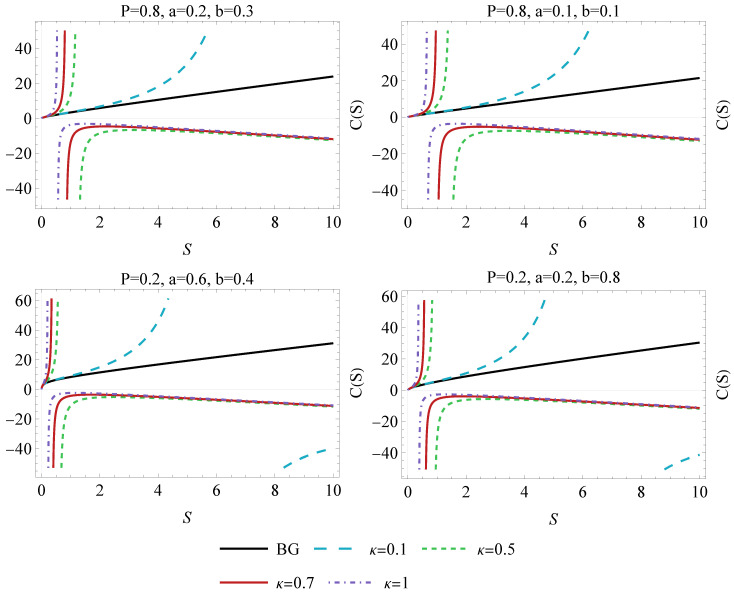
The heat capacity of a Van der Waals black hole as a function of entropy *S* for different values of κ, *P*, *a* and *b*. The black line corresponds to the BG characteristics of C(S).

The numerator of the expression, $C_{0}$, consists of terms dependent on κ, *a*, *b*, and *P*, reflecting the influence of Kaniadakis entropy, intermolecular forces, molecular pressure, and Λ on the heat capacity. Additionally, the complexity of the denominator underscores the intricate interplay between these parameters in shaping the heat capacity. The behavior of the heat capacity under the different values of κ is presented in Figure 3. Notably, a change in sign corresponds to the presence of divergent and physical limitation points, indicating a phase transition process, either a second-order or a first-order phase transition, respectively. Note that for the certain values of κ and *S*, the heat capacity indicates the local instability of a VdW BH. On the other hand, for κ→0, the BH eventually will be stable, as the C(S) diagram will approach the standard characteristics of the VdW BH [22,47].

### 3.2. P−V Criticality

The analysis of the critical behavior of the VdW BH within the Kaniadakis statistics regime can be carried out by using the P−V phase transitions. Notably, Van der Waals fluids are characterized by the P−T relationship given by:

(20) $$\begin{array}{c} P+\frac{a}{V^{2}}\left(V-b\right)=T, \end{array}$$

or

(21) $$\begin{array}{c} \frac{-ab+aV-TV^{2}}{V^{2}\left(b-V\right)}=P. \end{array}$$

In this part of the work, we will derive analogous equations for the VdW BH in the extended phase space of Kanidakis thermodynamics.

After some algebra, by using (9), (10) and (14), we obtain the relationship between the pressure and the horizon radius:

(22) $$\begin{array}{c} P\left(r_{h}\right)=\frac{T\cosh\left(\pi \kappa r_{h}\right)}{2(b+r_{h})}-\frac{a}{{\left(3b+2r_{h}\right)}^{2}}. \end{array}$$

In order to compare Equation (22) with the VdW relation (21), one can associate the horizon radius with the specific volume *V* [22]:

(23) $$\begin{array}{c} r_{h}=\frac{V-3}{b}. \end{array}$$

Then, the P(V) relationship within Kaniadakis framework is:

(24) $$\begin{array}{c} P\left(V\right)=-\frac{a}{V^{2}}-\frac{T\cosh\left(\frac{1}{4}\pi \kappa \left(V-3b\right)\right)}{b-V}, \end{array}$$

and for κ→0, the behavior of the Van der Waals fluid (21) is recovered.

In order to determine the qualitative behavior of critical points, we note that those points occur when P(v) has an infection point, i.e.,

(25) $$\begin{array}{c} \frac{\partial P}{\partial V}=0,\;\;\;\;\frac{\partial^{2}P}{\partial V^{2}}=0. \end{array}$$

Since Equation (21) can be expressed in the cubic form,

(26) $$\begin{array}{c} -ab+aV-bPV^{2}+PV^{3}-TV^{2}=0, \end{array}$$

the critical temperature, volume, and pressure can be obtained from comparison with the coefficients of the equation $P_{c}{\left(V-V_{c}\right)}^{3}=0$. To proceed further, we need to expand expression (24) according to the $cosh\left(x\right)=1+\frac{x^{2}}{2}+O\left(...\right)$. This leads to the following relation:

(27) $$\begin{array}{c} P\left(V\right)\approx \frac{-ab+aV-TV^{2}}{V^{2}\left(b-V\right)}-\frac{\kappa^{2}\left(\pi^{2}T{\left(V-3b\right)}^{4}\right)}{32(b-V)}+O\left(\kappa^{3}\right). \end{array}$$

Now, from the $P_{c}{\left(V-V_{c}\right)}^{3}=0$ and conditions (25), we can get the critical coefficients:

(28) $$\begin{array}{c} P_{c}=\frac{a}{27b^{2}},\;\;\;\;V_{c}=3b,\;\;\;\;T_{c}=\frac{8a}{27b}. \end{array}$$

Interestingly, they do not depend on the constant κ, coinciding with the coefficients of the Van der Waals fluid [18]. Hence, when BH behaves explicitly like the VdW fluid, the different entropies lead to effectively similar behavior in the P−V diagrams as we obtained using Bekenstein and Hawking laws [1,52]. Hence, our analysis partially supports arguments on thermodynamic inconsistencies, as was mentioned in [56]. In detail, it was shown therein that the choice of entropy does not really matter, since the results for all entropies coincide with the ones obtained from the usual Gibbs–Boltzmann statistics. Since the P(V) characteristic is independent of the form of entropy, for details on the characteristics for a VdW BH in a standard statistical regime, we refer to the [22,47].

### 3.3. Gibbs Free Energy

In order to obtain a complete description of the phase transition for the BH system we adopt a special thermodynamic potential. In this way, the Gibbs free energy is a thermodynamic quantity evaluated on the grounds of Euclidean action via a tailored limiting factor. A global stability analysis is carried out on the evidence of the sign of the Gibbs free energy. In the extended phase space, the thermodynamic potential is in turn the Gibbs free energy G=M−TS=H−TS. It is worth noting here that all discontinuous changes in the first- or second-order derivatives of the Gibbs energy induce a first- or second-order phase transition in the process. Hence, the Gibbs free energy can be defined as follows:

(29) $$\begin{array}{cc} G= & \frac{1}{6}\pi \left(\frac{a\left(-9b^{2}+18br_{h}+12r_{h}^{2}\right)}{3b+2r_{h}}-12ab\log\left(\frac{r_{h}}{b}+\frac{3}{2}\right)+4Pr_{h}^{2}\left(3b+2r_{h}\right)\right)\\ & -\frac{1}{\kappa }\frac{\frac{a\left(-9b^{2}+18br_{h}+12r_{h}^{2}\right)}{3b+2r_{h}}-12ab\log\left(\frac{r_{h}}{b}+\frac{3}{2}\right)+4Pr_{h}^{2}\left(3b+2r_{h}\right)}{12r_{h}^{2}}+\frac{3ab^{2}\left(3b+4r_{h}\right)}{4\left(3br_{h}+2r_{h}^{2}\right)}-\frac{2ab}{3br_{h}+2r_{h}^{2}}\\ & +ab\log\left(\frac{r_{h}}{b}+\frac{3}{2}\right)+bP+\frac{4Pr_{h}}{3}\mathrm{Sin}h\left[\pi \kappa r_{h}^{2}\right]. \end{array}$$

**Figure 4:**
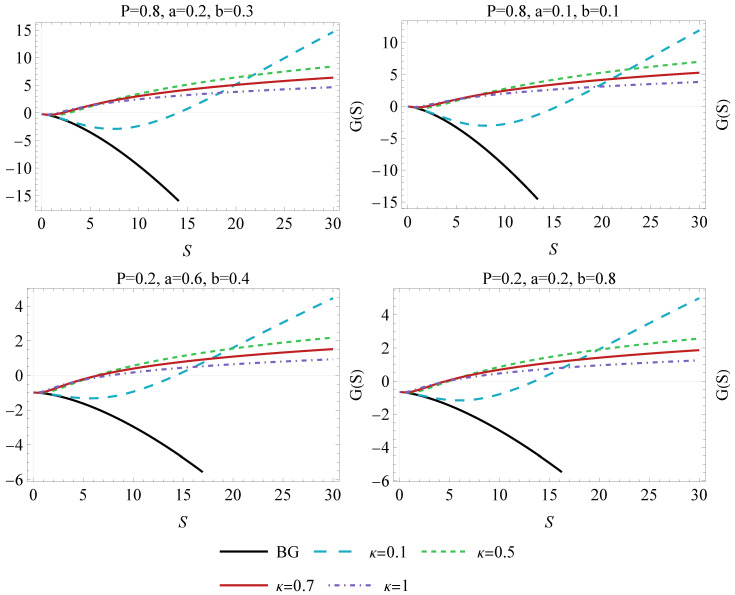
The Gibbs free energy diagram of a Van der Waals black hole as a function of entropy *S*, for different pressures *P*, constants *a*, and *b* varying values of κ vs. Boltzmann–Gibbs characteristic.

The functional behavior of the Gibbs free energy in the form of the G−S diagram has been shown in the Figure 4. Note that as κ increases, the curves become steeper, particularly at higher entropy values. This reflects that larger κ values tend to amplify the deviations from the usual thermodynamic behavior [8,22,47]. The analysis reveals that the G(S) in Van der Waals black holes is highly sensitive to the Kaniadakis entropy parameter κ and the Van der Waals parameters *a* and *b*. Negative values of Gibbs energy, particularly at small values of entropy, suggest the potential for phase transitions or thermodynamic instability. This effect is more pronounced for larger values of *a* and lower pressure. Additionally, as entropy increases, the system generally becomes more stable. However, strong interactions (larger *a*) and low pressure can lead to complex, potentially unstable, thermodynamic states at low entropy.

## 4. Energy Emission

Since BH radiates, the quantum fluctuations impose the creation of the particles and antiparticles, beyond the event horizon $r_{h}$. In fact, through the quantum tunneling phenomenon, particles with the positive energy may escape the innermost regions of Hawking radiation, giving rise to the evaporation process. The rate at which the black hole evaporates is dependent on its energy emission rate. This rate can be observed by an observer located far from the black hole. For such an observer, the black hole’s shadow corresponds to a high-energy absorption cross-section. This absorption cross-section characterizes how the black hole interacts with incoming radiation. In what follows, the limiting value of the cross-section is approximately [47,57]:

(30) $$\begin{array}{c} \sigma_{lim}\approx \pi r_{h}^{2}. \end{array}$$

Thus, the BH energy emission rate is given by:

(31) $$\begin{array}{c} \frac{d^{2}\epsilon }{d\omega dt}=\frac{2\pi^{2}\sigma_{lim}}{e^{\omega /T}-1}\omega^{3}, \end{array}$$

for the modified temperature *T* from Equation (14). Then, by using Equations (8) and (9), together with relation (30), one can obtain the emission rate as a function of $r_{h}$ and VdW parameters. Hence, the final expression for the energy emission is:

(32) $$\begin{array}{cc} \frac{d^{2}\epsilon }{d\omega dt} & =\frac{2\left(\pi^{3/2}b\kappa^{2}\left(a+9b^{2}P\right)\sqrt{\frac{\sinh^{-1}\left(\sinh\left(\pi \kappa {\mathrm{rh}}^{2}\right)\right)}{\kappa }}+4P\sinh^{-1}{\left(\sinh\left(\pi \kappa {\mathrm{rh}}^{2}\right)\right)}^{2}+\sinh^{-1}\left(\sinh\left(\pi \kappa {\mathrm{rh}}^{2}\right)\right)\right)}{\kappa^{2}\sqrt{\frac{\sinh^{-1}\left(\sinh\left(\pi \kappa {\mathrm{rh}}^{2}\right)\right)}{\kappa }}\sqrt{\pi \sinh^{2}\left(\pi \kappa {\mathrm{rh}}^{2}\right)+\pi }{\left(3\sqrt{\pi }b+2\sqrt{\frac{\sinh^{-1}\left(\sinh\left(\pi \kappa {\mathrm{rh}}^{2}\right)\right)}{\kappa }}\right)}^{2}}\\ & \times \pi a\kappa +21\pi b^{2}\kappa P+16\sqrt{\pi }b\kappa P\sqrt{\frac{\sinh^{-1}\left(\sinh\left(\pi \kappa {\mathrm{rh}}^{2}\right)\right)}{\kappa }}. \end{array}$$

**Figure 5:**
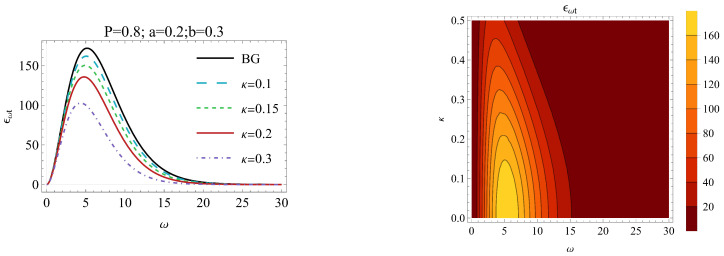
The energy emission rate as a function of ω for different choices of free parameters P,a,b and κ. The black line corresponds to the standard entropy from the Boltzmann–Gibbs (BG) statistics.

The behavior of the energy emission rate as a function of ω for different choices of κ is presented in Figure 5. One can see that there exists a peak of the energy emission rate for the VdW black hole. Clearly, the introduction of the Kaniadakis entropy dampens the emission rate and lowers this peak. This means that under Kaniadakis statistics, at least at the beginning of the process, the black hole evaporates in a slower pace. However, as the ω increases, the effect of the κ-statistics is indistinguishable from the behavior associated with the Boltzmann–Gibbs formula. Hence, at the regimes of the high ω, the choice of the entropy (10) is not significant.

## 5. Summary and Conclusions

In this work, we investigated the thermodynamics of the Van der Waals black hole within the framework of relativistic Kaniadakis entropy. Our analysis reveals that the Kaniadakis parameter κ significantly alters the thermodynamic properties of the black hole, particularly its mass, temperature, and Gibbs free energy, as functions of entropy. These modifications suggest the presence of thermodynamic instabilities at low entropy, with the parameters *a* and *b* of the Van der Waals model further influencing these effects.

Interestingly, our findings show that the pressure–volume P−V behavior of the Van der Waals black hole remains independent of the chosen entropy form, aligning with the results obtained before by Ditta and colleagues in [47]. This observation aligns with previous research suggesting that, under specific conditions, nonadditive entropic models are equivalent to those based on standard Boltzmann–Gibbs thermodynamics [56]. Thus, the form of entropy appears to have minimal impact on the critical behavior of the system. Additionally, we found that Kaniadakis entropy affects the energy emission rate of the black hole, with the κ parameter leading to a slower evaporation rate during the early stages of the process. However, at higher energies, the emission rate converges with predictions obtained from Boltzmann–Gibbs statistics [47], indicating that the influence of Kaniadakis entropy is only significant in low-energy regimes.

Overall, our results constrain the flexibility of the Van der Waals black hole model in accommodating nonadditive statistical frameworks, since some BH properties, such as the P−V relationship or energy emission rates, are preserved. This study paves the way for further investigation on the role of generalized entropies in black hole thermodynamics and their implications for understanding black holes. Referring to the “entropic cosmology inconsistency” argument from [56], which states that *with consistent thermodynamic quantities defined on the Hubble horizon, satisfying the Clausius relation, and employing a linear M−L relation, all nonadditive entropic force models are identical to those derived from the standard Bekenstein entropy and Hawking temperature*, the Van der Waals black hole can serve as a valuable testing ground for the nonadditive entropies, such as Kaniadakis, Renyi, Tsallis etc. [25]. Thus, future work should focus on an exploration of the phase transitions and the Clausius relation in different statistical regimes for VdW BH, potentially gaining more insights on the consistency (or the lack thereof) nonadditive entropies [58]. In this context, the viability of statistics other than the Boltzmann–Gibbs form for black hole thermodynamics remains a topic of debate [59].

## Data Availability

The original contributions presented in the study are included in the article, further inquiries can be directed to the corresponding author.
